# Contrast-induced acute kidney injury: the importance of diagnostic criteria for establishing prevalence and prognosis in the intensive care unit

**DOI:** 10.5935/0103-507X.20170041

**Published:** 2017

**Authors:** Edmilson Leal Bastos de Moura, Fábio Ferreira Amorim, William Huang, Marcelo de Oliveira Maia

**Affiliations:** 1 Intensive Care Unit, Hospital Santa Luzia - Brasília (DF), Brazil.; 2 Escola Superior de Ciências da Saúde - Brasília (DF), Brasil.

**Keywords:** Contrast media/adverse effects, Acute kidney injury/chemically induced, Renal dialysis, Severity of illness index, Risk assessment, Prognosis

## Abstract

**Objective:**

To establish whether there is superiority between contrast-induced acute kidney injury and contrast-induced nephropathy criteria as predictors of unfavorable clinical outcomes.

**Methods:**

Retrospective study carried out in a tertiary hospital with 157 patients undergoing radiocontrast infusion for propaedeutic purposes.

**Results:**

One hundred forty patients fulfilled the inclusion criteria: patients who met the criteria for contrast-induced acute kidney injury (59) also met the criteria for contrast-induced nephropathy (76), 44.3% met the criteria for KDIGO staging, 6.4% of the patients required renal replacement therapy, and 10.7% died.

**Conclusion:**

The diagnosis of contrast-induced nephropathy was the most sensitive criterion for renal replacement therapy and death, whereas KDIGO showed the highest specificity; there was no correlation between contrast volume and progression to contrast-induced acute kidney injury, contrast-induced nephropathy, support dialysis or death in the assessed population.

## Abstract (pt)

**Objetivo:**

Estabelecer se há superioridade entre os critérios para predizer desfecho clínico desfavorável na lesão renal aguda e nefropatia induzidas por contraste.

**Métodos:**

Estudo retrospectivo conduzido em hospital terciário com 157 pacientes submetidos à infusão de contraste radiológico para fins propedêuticos.

**Resultados:**

Cumpriram os critérios para inclusão 147 pacientes. Aqueles que cumpriram os critérios de lesão renal aguda induzida por contraste (59) também cumpriram os critérios para nefropatia induzida por contraste (76); 44,3% dos pacientes cumpriram os critérios para o estadiamento pelo sistema KDIGO; 6,4% dos pacientes necessitaram utilizar terapia de substituição renal, e 10,7% dos pacientes morreram.

**Conclusão:**

O diagnóstico de nefropatia induzida por contraste foi o critério mais sensível para determinar a necessidade de terapia de substituição renal e óbito, enquanto o KDIGO demonstrou a maior especificidade; na população avaliada, não houve correlação entre o volume de contraste e a progressão para lesão renal induzida por contraste, nefropatia induzida por contraste, diálise de suporte ou óbito.

## INTRODUCTION

Contrast-induced acute kidney injury (CIAKI) is an important cause of in-hospital acquired renal failure, surpassed only by diseases that cause renal hypoperfusion and the use of nephrotoxic drugs.^(1)^ This entity has other names, with contrast-induced nephropathy (CIN) being the most well-known. Contrast-induced acute kidney injury or CIN is described as the sudden worsening of renal function after the administration of intravenous contrast after ruling out other known causes.^(2)^

**Figure 1:**
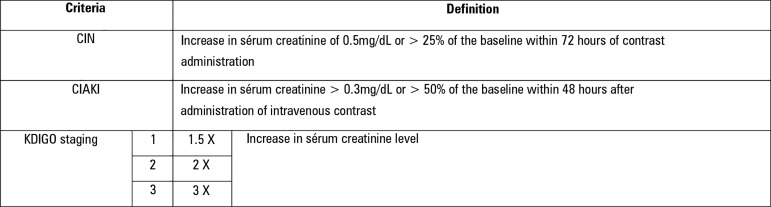
Definition of contrast-induced nephropathy, contrast-induced acute kidney injury and Kidney Disease: Improving Global Outcomes staging criteria. CIN - contrast-induced nephropathy; CIAKI - contrast-induced acute kidney injury; KDIGO - Kidney Disease: Improving Global Outcomes. Adapted from American College of Radiology,^(3)^ Mehta et al.,^(4)^ Mehran et al.,^(5)^ Barrett et al.,^(6)^ e KDIGO Clinical Practice Guideline for Acute Kidney Injury.^(10)^

The definition of this disease entity is not uniform, with the used criteria showing discrepancies. According to the Acute Kidney Injury Network (AKIN), CIAKI is defined as an increase in serum creatinine > 0.3mg/dL or > 50% of the baseline within 48 hours after the administration of intravenous contrast (Figure 1).^(3,4)^ As many physicians who address this condition are radiologists, the European Society of Urogenital Radiology defines kidney injury as CIN (well received by the radiological community) if there is an increase in serum creatinine of 0.5mg/dL or > 25% of the baseline within 72 hours of contrast administration^(3,5-8)^ in the absence of an alternative etiology. These are arbitrary, laboratory testing-based definitions that are useful for statistical comparisons in clinical trials,^(9)^ and both definitions are widely used.^(3-7)^ Such divergence has a direct impact on prevalence assessments, as the use of different criteria changes the observed results.

Equally important for the definition of acute kidney injury is the use of the Kidney Disease: Improving Global Outcomes (KDIGO) staging, a guideline proposed in 2012,^(10)^ which supports the classification of acute kidney injury. However, this diagnostic tool covers acute kidney injury (AKI) of any etiology. There is no study that has correlated this tool to the CIAKI and CIN diagnostic criteria.

Considering its prevalence and clinical importance, preventing the onset of kidney injury would be ideal. However, due to the ineffectiveness of prophylactic measures, especially in critically ill patients, as well as the difficulty in establishing specific biomarkers for its identification, early diagnosis might be an option for successful treatment. It is noteworthy that there is no comparative study of the diagnostic criteria, and, therefore, there is no uniformity of information available in the medical literature.

In this context, we suggest a comparative study of the abovementioned diagnostic criteria and a comparison between these criteria and the KDIGO staging, carried out in patients admitted to the intensive care unit (ICU), to promote the discussion of this issue, to identify a possible diagnostic or prognostic superiority between them and to correlate them to the KDIGO score.

The objectives of this study were as follows: to determine the prevalence of CIAKI, CIN and the KDIGO staging score classification in critically ill patients; to determine if there was an association between CIN, CIAKI and KDIGO with an adverse outcome (renal replacement therapy - RRT or death); to determine if there was a correlation between the diagnosis of CIAKI, CIN and the KDIGO staging score; and to determine if there was a correlation between the volume of radiocontrast, the diagnosis of CIAKI, CIN, the need for hemodialysis or death.

## METHODS

The patients selected for the study were admitted to the ICU of *Hospital Santa Luzia*, Brasília - Distrito Federal, from November 2012 to February 2014. All patients were subjected to volume expansion with 0.9% saline crystalloid solution (for CIN prophylaxis purpose) and none received intravenous sodium bicarbonate or N-acetylcysteine prior to contrast use.

As mentioned above, CIAKI was defined as an increase in serum creatinine > 0.3mg/dL or > 50% of the baseline within 48 hours after administration of intravenous contrast; CIN was defined as an increase in serum creatinine of 0.5mg/dL or > 25% of the baseline within 72 hours of contrast administration in the absence of an alternative etiology.

Epidemiological data collected from medical records were as follows: sex, age, Acute Physiological and Chronic Health Evaluation II (APACHE II), Simplified Acute Physiology Score (SAPS II) and Sequential Organ Failure Assessment (SOFA) scores, main and secondary diagnoses, day of hospital admission when the test was performed and the segment that was assessed, type and volume of contrast used, basal value of serum creatinine in the first three days after contrast use (in case of more than one collection on the same day, the highest value was considered) and the highest value obtained during the ICU stay, day of hospital admission day when this value was obtained, and KDIGO staging related to the first three days after contrast use and admission score. Additionally, weight [according to the adjusted weight formula = (current weight - ideal weight) x 0.25 - (current weight), where Ideal weight = 24.9 x height^2^] and height (using the half-scale measurement) were estimated.

Inclusion criteria were as follows: patients subjected to examinations using contrast media, regardless of age, with an ICU length of stay longer than three days. Exclusion criteria were prior history of allergy to iodinated contrast, previous diagnosis of nephropathy or RRT, advanced disease with limited therapeutic efforts, length of stay less than 3 days in the ICU (due to ICU discharge or death), patients subjected to multiple contrast studies during the same hospital stay, absence of a baseline serum creatinine value, lack of consent from the patient or patient's legal representative for contrast administration.

The basal creatinine was obtained from the patient electronic records or from previous examinations, always considering the lowest value obtained in the 12 months prior to hospital admission. Based on this value, which was considered the baseline value, creatinine levels on the first three days were collected after the use of intravenous contrast, as well as the highest value recorded during ICU stay. These data allowed us to define the KDIGO staging score.

The iodinated contrast agent used was Optiray^®^ 320 (low osmolality, 702mOsm/kg, viscosity 5.8 at 37ºC) containing ioversol (320mg/mL of iodine, non-ionic monomer, Mallinckrodt^®^). The volume used intravenously was 1.3mL/kg, according to criteria established by *Hospital Santa Luzia* Imaging Diagnostic Center. Using this information, it was possible to establish the ratio between the contrast volume and the patient's corrected weight (in mL/kg).

Statistical analysis was performed using Statistical Package for Social Science (SPSS), version 20. We used the χ-square test, Kappa index of agreement, Student's *t* test and Levene test for the assessment of homogeneity (equality) of population variances.

The study was authorized by the Research Ethics Committee of *Hospital das Forças Armadas* (HFA - Brasília, Distrito Federal). The free and informed consent was waived due to the retrospective and observational nature of the study.

## RESULTS

**Figure 2:**
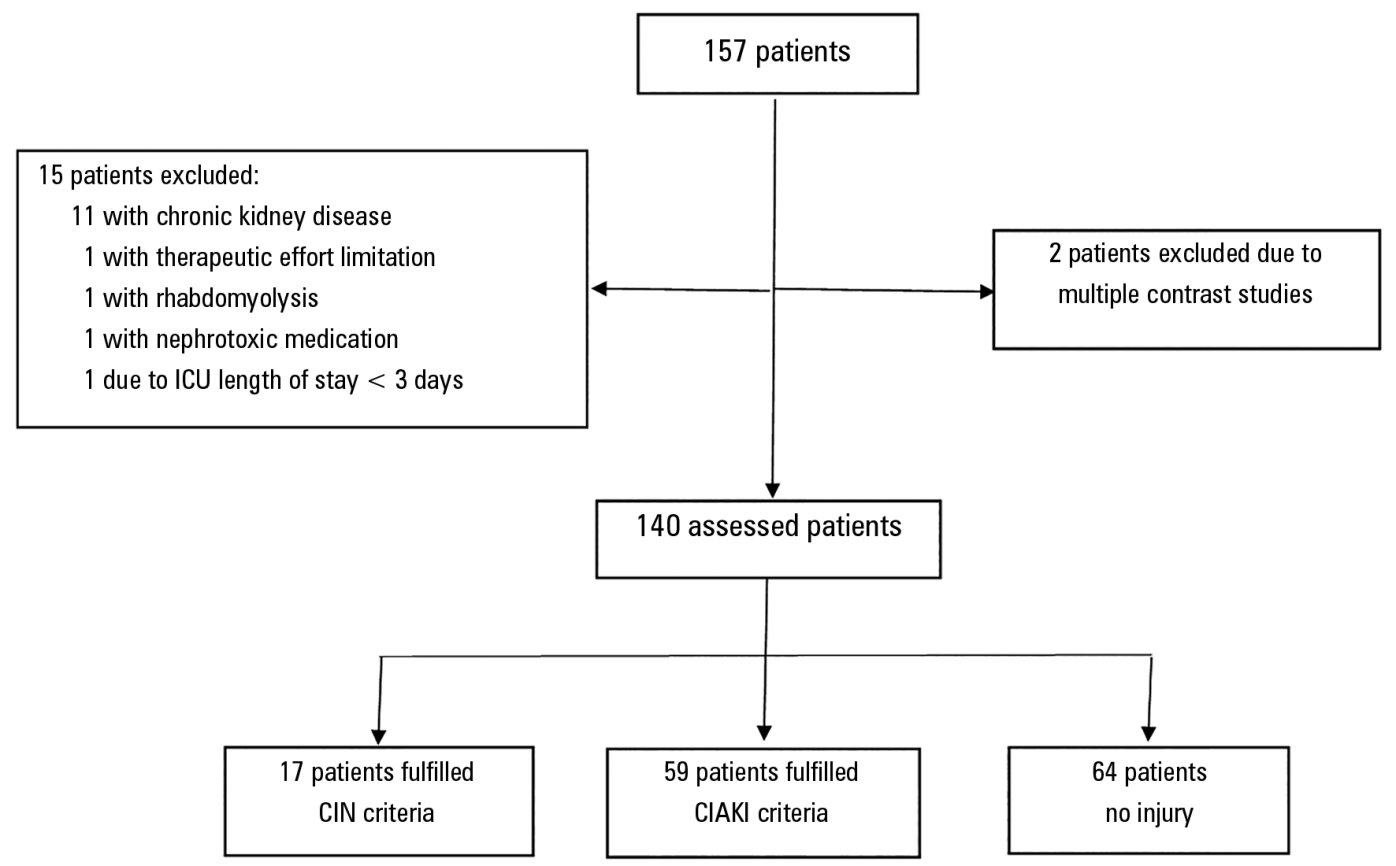
Study design. Initial population sample, excluded patients and the division into two groups. ICU - intensive care unit; CIN - contrast-induced nephropathy; CIAKI - contrast-induced acute kidney injury.

One hundred fifty-seven patients were assessed, of which 17 individuals were excluded, two due to multiple tests at the same hospital admission, 11 due to history of chronic renal failure, one due to nephrotoxic medication, one due to rhabdomyolysis, one due to ICU length of stay < 3 days and one patient that had limited therapeutic efforts (Figure 2).

Data collected from the studied population are summarized in table 1. The most prevalent causes of ICU admission in this population were pulmonary thromboembolism (15.7%), community acquired pneumonia (10%) and ischemic stroke (5.7%). Table 2 shows the distribution of the studied population according to their classification in the three scores (CIAKI, CIN and KDIGO), as well as progression to RRT or death. There were no patients classified using only the CIAKI criteria; all patients who met these diagnostic criteria were also classified using the CIN, totaling 59 individuals, corresponding to 42.1% of the sample; all the patients who were classified in the CIAKI group also belonged to the CIN group, but the inverse relationship was not observed. Nine patients (6.4% of the sample) required RRT support during the ICU stay, and fifteen patients (10.7%) died.

**Table 1:** Epidemiological data and severity scores in the assessed population

	Mean	Median	SD
Age (years)	60.8	62.0	21.1
APACHE II	9.9	9.0	6.0
SAPS II	30.1	31.0	11.5
SOFA	1.8	1.0	2.7
Contrast volume (mL)	92.9	90.0	10.3
ICU stay (days)	10.5	7.0	10.3
Basal creatinine (mg/dL)	0.77	0.76	0.26

SD - standard deviation; APACHE II - Acute Physiological and Chronic Health Evaluation; SAPS II - Simplified Acute Physiology Score; SOFA - Sequential Organ Failure Assessment; ICU - intensive care unit.

**Table 2:** Classification and kidney injury scores in the studied population

Criteria	N = 140 N (%)	LOS ICU (days)	Mortality N (%)	RRT N (%)
Contrast-related				
CIAKI	59 (42.1)	11.5	9 (6.5)	7 (5)
CIN	17 (12.1)	11.6	2 (1.4)	1 (0.7)
No CIN/CIAKI criteria	64 (45.8)	8.9	4 (2.8)	1 (0.7)
KDIGO staging				
No KDIGO criteria	78 (55.7)	7.9	0	1 (0.7)
Stage I	33 (23.5)	9.3	2 (1.4)	0
Stage II	12 (8.5)	13.8	2 (1.4)	0
Stage III	17 (12.1)	22	11 (7.9)	8 (5.7)
			15 (10.7)	9 (6.4)

LOS - length of stay; ICU - intensive care unit; RRT - renal replacement therapy; CIAKI - contrast-induced acute kidney injury; CIN - contrast-induced nephropathy; KDIGO - Kidney Disease: Improving Global Outcomes. The CIN/CIAKI group included patients with both classifications; The CIN group included patients with only this classification.

Table 3 shows the risk assessment, sensitivity, specificity, positive and negative predictive values, and odds ratio of each diagnostic criterion for progression to RRT or death. Table 4 is a descriptive table of concordance (using Kappa index) between kidney injury scores.

**Table 3:** Values of relative risk of progression to hemodialysis and death according to the classification in the different kidney injury scores

	CIAKI		CIN		KDIGO	
	*Versus* RRT	*Versus* death	*Versus* RRT	*Versus* death	*Versus* RRT	*Versus* death
Relative risk	4.8 (1.0 - 22.3)	2.0 (0.7 - 5.4)	6.7 (0.8 - 52.4)	2.3 (0,7 - 6.9)	5.0 (1.5 - 17.3)	2.3 (0.8 - 6.5)
Sensitivity (%)	77.7 (45.2 - 93.6)	60 (35.7 - 80.1)	88.8 (56.5 - 98)	73.3 (48.0 - 89.1)	44.4 (18.8 - 73.3)	26.6 (10.9 - 51.9)
Specificity (%)	60.3 (51.7 - 68.2)	60 (51.2 - 68.1)	48 (39.7 - 56.5)	48 (39.4 - 56.6)	88.5 (81.9 - 92.9)	88 (81.1 - 92.5)
Positive predictive value	1.9 (1.3 - 2.9)	1.5 (0.9 - 2.3)	1.7 (1.2 - 2.2)	1.4 (1.0 - 2.0)	3.8 (1.6 - 9.2)	2.2 (0.8 - 5.8)
Negative predictive value	0.3 (0.1 - 1.2)	0.6 (0.3 - 1.2)	0.2 (0.04 - 1.4)	0.5 (0.2 - 1.3)	0.6 (0.3 - 1.1)	0.8 (0.6 - 1.1)
Odds ratio of diagnosis	5.3 (1.0 - 26.6)	2.2 (0.7 - 6.7)	7.4 (0.9 - 60.9)	2.5 (0.7 - 8.4)	6.1 (1.5 - 25.6)	2.6 (0.7 - 9.4)

CIAKI - contrast-induced acute kidney injury; CIN - contrast-induced nephropathy; KDIGO - Kidney Disease: Improving Global Outcomes; RRT - renal replacement therapy. Values within the 95% confidence interval are shown between parentheses.

**Table 4:** Concordance between kidney injury scores

	CIAKI *versus* CIN	CIAKI *versus* KDIGO	CIN *versus* KDIGO
Concordance (%)	87.9	71.4	59.3
Kappa index	0.7	0.3	0.2
p value	0.000	0.000	0.000

CIAKI - contrast-induced acute kidney injury; CIN - contrast-induced nephropathy; KDIGO - Kidney Disease: Improving Global Outcomes. Results expressed in %. Kappa index in punctuation.

The analysis of the correlation between contrast volume and the diagnosis of CIAKI, CIN, the need for RRT or death outcome is summarized in table 5. The relative risk of the RRT outcome was statistically significant in the groups classified using the CIAKI and KDIGO staging scores.

**Table 5:** Correlation between the volume of contrast used and the onset of kidney injury, need for renal replacement therapy (hemodialysis) and death in the studied population

	p value
Contrast volume *versus*	
CIAKI	0.138
CIN	0.189
RRT	0.44
Death	0.62

CIAKI - contrast-induced acute kidney injury; CIN - contrast-induced nephropathy; RRT - renal replacement therapy.

The relative risk of death was not statistically significant in any group. For the RRT and death outcomes, the sensitivity was higher in the CIN group. The specificity, positive and negative predictive values were higher in the KDIGO group. Considering only the CIAKI and CIN groups, specificity, positive and negative predictive values were higher in the CIAKI group.

## DISCUSSION

The results obtained in this study showed that for the diagnoses of CIAKI, CIN or KDIGO staging criteria were risk factors for RRT or death. In this population, the diagnosis of CIN was the most sensitive criteria for RRT and death, whereas KDIGO showed the highest specificity. The higher CIN sensibility, compared to CIAKI, might be due to the longer period that is considered for the diagnosis of AKI (72 *versus* 48 hours). The ICU in which our patients were admitted focuses on a wide range of medical issues (except for coronary heart disease and surgical patients), and the most prevalent diagnoses were pulmonary thromboembolism, severe community acquired pneumonia and ischemic stroke. Considering that, in some cases, institutional protocols determine ICU admission, regardless of the disease severity, patients with an APACHE score below the general unit average were selected.

The importance of CIAKI in the ICU is indisputable, and its prevalence is confirmed by the results of the present study (CIAKI: n = 59, 42.1%, CIN: n = 17, 12.1%). However, the guidelines that support current medical practice are not specific to the critical patient,^(10-12)^ justifying studies aimed at this population.

Critically ill patients have other concomitant risk factors for the onset of CIAKI, such as hypovolemia, congestive heart failure, diabetes, age > 70 years and the use of nephrotoxic drugs (e.g., aminoglycosides, vancomycin, amphotericin B, nonsteroidal anti-inflammatory drugs). These factors make this population particularly susceptible to this condition. In this study, one patient was excluded from the study due to the concomitant use of nephrotoxic drugs, which would confound the analysis of radiocontrast as a causal factor of the AKI.

The identification of factors and biomarkers that predict the risk of kidney impairment following contrast studies are a popular topic. Patients at risk for developing CIAKI or CIN could be mostly identified through questionnaires. Values are assigned to certain risk factors, establishing risk-prediction scores^(13)^ of dialytic support and mortality in one year. The use of protocols directed at the early detection of critically ill patients at risk is equally important.^(14)^

Basal creatinine, used as an individual parameter of normality in our study, has great prognostic importance for the onset of CIN, ranging between 2% (for values < 1.5) to 20% (for values > 2.5mg/dL).^(15)^ Creatinine levels, which are a filtration marker and indirectly measure kidney injury, have important limitations, such as low sensitivity. The development of more accurate biomarkers should help identify patient subpopulations at high risk of developing severe AKI.^(16)^

In a retrospective study carried out by Ledermann et al.,^(17)^ in which a questionnaire was applied, only 45% of the population of 1,766 outpatients (with positive risk factors such as kidney disease, renal surgery, use of nephrotoxic drugs) needed serum creatinine measurements. In critically ill patients, this approach would be very different, considering that most of these patients would have risk factors due to their comorbidities. Therefore, perhaps an adaptation of the questionnaires is required for use in patients admitted to ICU.

The correlation between the amount of radiological contrast administered to the patient and CIAKI has been acknowledged for some time.^(18)^ We observed the routine use of simple rules to determine the volume to be infused, using amounts that are well below those considered maximum amounts.^(19)^ This practice, however, does not prevent the onset of kidney injury, as demonstrated in this study (prevalence of CIAKI: 42.1%). It could be affirmed that there is no statistically significant correlation between the contrast volume and the diagnosis of CIAKI, CIN, need for RRT or death in this study.

Contrast-induced nephropathy recovery could take at least days (to weeks) to be completed (with potential sequels).^(20)^ We considered as an exclusion criterion the fact of being subjected to multiple contrast studies in the same hospital, independent of the time interval between tests. This strategy aimed at excluding patients who had accumulative contrast excess. However, invasive imaging workup has been a widely used resource in the ICU, and this excess of radiocontrast agent should be taken into account as a causal factor in the growing incidence of CIAKI.

It is vital to consider measures to prevent the onset of CIAKI. The first step is always to discuss and consider the benefit of performing the contrast study with patients or legal representatives. The most effective prophylaxis appears to be intravenous hydration, regardless of the type of fluid used,^(2)^ although there is evidence in favor of 0.9% saline solution use.^(21)^ Extracellular volume expansion using an intravenous sodium bicarbonate solution^(22)^ and the use of low contrast volume (isomolar type) are other measures that have been shown to reduce the risk of CIN.^(7)^ The use of N-acetylcysteine was not effective in preventing CIN.^(23)^

We acknowledge the absence of multivariate analysis and the population size as a limitation of our study. Studies involving a larger number of individuals, where such analysis is not impaired by the sample, will bring us new answers.

As it could be observed, there is no consensus on the nomenclature that defines this complication or even on which criterion appears to be more clinically significant. Comparatively, the criteria for the diagnosis of CIN appear to be less stringent than those needed for the diagnosis of CIAKI. It is noteworthy that there was no diagnosis of CIAKI without a concomitant inclusion in the CIN criteria, supporting the concept that the second criterion is more comprehensive.

Whatever criterion is used to establish the diagnosis should be regarded as a tool that encourages early identification and intervention in this condition, decreasing the harmful impact on critically ill patients.

## CONCLUSION

A higher number of patients with acute kidney injury was identified when using the contrast-induced nephropathy criterion. Diagnosis of contrast-induced nephropathy was the most sensitive criteria for renal replacement therapy and death, whereas KDIGO showed the highest specificity. Also there was no correlation between contrast volume and progression to contrast-induced acute kidney injury, contrast-induced nephropathy, renal replacement therapy for dialytic support or death.
